# Effects of emission trading schemes on corporate carbon productivity and implications for firm-level responses

**DOI:** 10.1038/s41598-021-91193-4

**Published:** 2021-06-03

**Authors:** Hail Jung, Seyeong Song, Young-Hwan Ahn, Ha Hwang, Chang-Keun Song

**Affiliations:** 1 Ulsan National Institute of Science and Technology, Ulsan, 44919 Republic of Korea; 2 Sookmyung Women’s University, Seoul, 04310 Republic of Korea; 3 The Korea Institute of Public Administration, Seoul, 03367 Republic of Korea

**Keywords:** Climate change, Climate-change policy

## Abstract

Since the South Korean government enacted the Emission Trading Scheme (ETS), companies have been striving to simultaneously improve productivity and reduce carbon emissions, which represent conflicting goals. We used firm-level emissions and corporate variables to investigate how ETS enactment has affected carbon productivity, which is a firm-level revenue created per unit of carbon emission. Results showed that firm-level carbon productivity increased significantly under the ETS, and such a trend was more evident for high-emission industries. We also found that companies with high carbon productivity were (1) profitable, (2) innovative, and (3) managed by CEOs with experience in environmental fields. These findings suggest that to achieve the conflicting goals of increasing corporate profits while reducing emissions, firms have to invest in green technologies, and such decisions are supported by green leadership. Our findings also have implications for corporate leadership; data highlight the importance of managing human resources and deploying investment policies to respond to ETS.

## Introduction

Global warming has progressed to the point of being both a serious environmental problem and socioeconomic problem, with a significant impact on business activities and public health^1,2^. Governments worldwide have enacted laws and regulations to reduce the effects of greenhouse gases (GHGs) such as carbon dioxide (CO_2_), methane (CH_4_), nitrous oxide (N_2_O), hydrofluorocarbons (HFCs), perfluorocarbons (PFCs), and sulfur hexafluoride (SF_6_). For instance, in 2019, the European Union (EU) announced its Green Deal, which aims to create a carbon–neutral society by 2050. Similarly, China has announced that it aims to achieve carbon neutrality by 2060. South Korea is also hoping to achieve this goal by 2050. On July 14, 2020, South Korea disclosed a detailed plan for its Korean New Deal initiatives, in which the government aims to achieve an economic revival through investment in clean energy^3^.

Furthermore, the COVID-19 crisis offers an opportunity for countries to awaken to the seriousness of known–unknown risks such as those posed by environmental issues, especially the effects of climate change. This is illustrated by recent movements that require companies to report environmental management activities. For example, the International Financial Reporting Standard Foundation has recently published a consulting paper that addresses the importance of establishing standards for disclosures of environmental-related information^4^. In South Korea, the Financial Supervisory Service (corresponding to the Securities and Exchange Commission (SEC) of the United States) has established a strategy for prompting the disclosure of sustainability management reports consisting of information on environmental, social and governance (ESG) activities, and the submission of ESG reports will be mandatory for all Korea Exchange-listed firms from 2030. From a firm’s perspective, it is important to formulate policies that can successfully lead to an era of sustainable growth in a decarbonized economy. Reducing firm-level emissions but not decreasing the firm’s productivity is fundamental.

In academia, research has tended to focus on CO_2_ as this GHG caused by human of total GHG emissions worldwide and is a primary driver of climate change^5,6^. Policies, therefore, have long focused on methods to reduce carbon emissions effectively. Specifically, as firms’ production activities are closely related to emissions, regulators have proposed policies that do not adversely affect corporate productivity while reducing carbon emissions.

One suggested approach to GHG reductions in industries is the enactment of emissions trading schemes (ETSs). These are based on a cap-and-trade mechanism in which emitters are allowed a certain level of emissions (the cap) and can buy or sell allowances with other firms (the trade) to maintain their emissions within the given allowances. Firms participating in the ETS make strategic choices to optimize performance, ideally by reducing emission levels while maintaining or increasing production levels. Following such global efforts, in 2010, the South Korean government enacted the Framework Act on Low Carbon, Green Growth. The Act consists of several emission reduction schemes such as the Energy and GHG Target Management System and the ETS. Both methods regulate and require the disclosure of emission details of firms exceeding annual emissions of 125,000 t CO_2_-eq (or those exceeding annual emissions of 25,000 t CO_2_-eq at a single installation). The former—the Energy and GHG Target Management System—began in 2011, while the latter—South Korea’s national-level ETS—began in 2015. Such government disclosures of emission details before the 2015 ETS enactment provide an interesting research setting that can allow researchers to pinpoint the before and after effects of ETS adoption by using emission data provided under the Energy and GHG Target Management System and those data gathered following the initiation of ETS laws, respectively.

Around 300 South Korean companies, which accounted for 60–70% of the country’s GHG emissions, participated in the first phase of the ETS in 2015. These firms were obliged to submit certified emission reports at the end of the commitment year. To monitor these firms and certify their emissions, the government designated 17 verified authorities and one accredited authority, the National Institute of Environmental Research. Currently, South Korea’s ETS is designed to follow three phases. The first phase (2015 to 2017) aimed to accumulate experience and stabilize the ETS in the country. This phase mainly was implemented to enhance flexibility and establish infrastructure for precise measurements, reporting, and verification (MRV). All emission allowances were allocated free of charge during the first phase. In the second phase (2018 to 2020), the objective was to significantly reduce GHGs by broadening the range of ETS participating firms and enhancing the emissions reporting quality and identification standards. Finally, the third phase (2021 to 2025) aims to achieve significant emission reductions by encouraging firms’ voluntary participation while allowing third parties to join the ETS to expand the supply of liquidity. To further constrain firm-level emissions, ETS participating firms must pay 10% of the initial carbon emission reduction cap set by the government. If firms fail to supplement their shortfall through trading certified emission reductions and exceed their allocated allowances, they will be fined ~ 100,000 KRW per 1 t CO_2_-eq. On the other hand, firms and installations with surplus allowances can sell them or transfer them to the next year.

The ETS is generally considered effective^7,8^. As a market-based mechanism that controls emissions by providing incentives for reductions, it is cost-effective with minimum social costs, thus enabling firms to develop their mitigation strategies to cope with climate change through free trade between businesses. Since its first introduction, researchers have regularly suggested that the ETS plays or will play a significant role in achieving a low-carbon society and promoting green growth^9^. In the EU, a growing body of research has examined post-ETS effects^10–13^, with many researchers finding that an ETS positively affects corporate performance^14–19^. For example, firms participating in the EU-ETS market were generally more profitable than those not participating^14^, and the system positively affected material costs for the power industry^18^. In addition, there was a positive association between emission abatement and trading profits^19^. Similarly, China’s ETS has induced firms to be innovative, thereby increasing the number of low-carbon-related patents being registered^20^.

In this respect, carbon productivity—a firm-level revenue generated by the carbon emissions—has become an important way to measure low-carbon development, as it integrates the goals of economic development and carbon reductions^21^. For example, on the national scale, China’s carbon productivity was positively affected by the gross domestic product (GDP) per capita, technology level, trade openness, and foreign direct investments while negatively affected by the energy consumption structure, industrial proportion, and urbanization level^22^. At the regional scale, an analysis of correlations between environmental regulation and carbon productivity in China showed that eastern China’s average intensity of environmental regulation was closest to its threshold, while those of central and western China were far from the threshold^23^. Similarly, an analysis of foreign trade’s effects on China’s carbon productivity showed a clear positive spatial spillover effect in which export–import activity significantly improved China’s carbon productivity (mainly through imports). In contrast, foreign trade in western China contributed the most to China’s increased carbon productivity but not in eastern and central China^21^. At the industry level, China’s pilot ETS boosted carbon productivity overall while being more effective in the petrochemical and electric power industries and less so in the building materials and transportation industries^24^.

While regional- and industry-level analyses of carbon productivity are increasing, examinations of the firm-level effects of carbon productivity during the ETS have been limited to our knowledge. This is because firm-level energy consumption or emission data before ETS implementation is difficult to obtain. Firm-level energy consumption and CO_2_ emissions data are challenging to collect, so these data are often not even recorded^25^. However, the South Korean government’s provision of firm-level emission reports starting in 2011, before ETS implementation in 2015, allows for the analysis of ETS effects on firms’ carbon productivity decisions. Investigating variations in carbon productivity is important because energy procurement strongly affects production costs. Furthermore, as each firm’s management group determines the amount of energy used and selects energy sources, energy-use data can reflect corporate strategy, including external factors such as environmental policies and energy prices^25^.

In this study, we tested how the relationship between ETS participation and carbon productivity was affected by industry-level emissions. We calculated individual firms’ carbon productivity by using the South Korean institutional data, and we applied a difference-in-difference model to investigate the effects of the ETS on carbon productivity. To employ the model, we sorted ETS participating firms and those that did not. Second, we classified high-pollution industries. Reducing emissions through the ETS is an especially significant concern for high-polluting industries as these firms in such industries are likely to be more directly affected by ETS participation. For example, in 2017, the basic metals manufacturing industry emitted over 123 million tCO_2_ while the car manufacturing industry emitted only 7 million tCO_2_. Such industry-level emission differences may imply differences in the effects of the ETS at the firm-level. Firms that belong to relatively less-polluting industries may find it more costly to lower GHGs than firms belonging to high-polluting industries.

We then tested how the relationship between ETS participation and carbon productivity was affected by firm-level emissions, while measuring carbon productivity as revenue generated scaled by carbon emissions (i.e., how much revenue a firm could generate by emitting a unit of CO_2_). To our knowledge, this study is the first attempt to examine differences in corporate responses before and after ETS enactment. We assessed how manufacturing firms adapted to the ETS phases based on corporate profitability, patent quantity, and board diversity to identify effective approaches used by firms to increase their carbon productivity. In this respect, a key contribution of this research is in empirically showing that the introduction of the ETS had positive effects on firms’ carbon productivity.

## Results

Changes in carbon productivity at the industry level (Table 1) showed that the severity of emission management might differ depending on business classification, even among the manufacturing industry. For the three highest-emission industries, carbon productivity significantly increased after participation in the ETS, an encouraging result as these industries account for most of South Korea’s emissions. On the other hand, industries that were less sensitive to emissions did not consistently react to ETS implementation. We found that some industries experienced an increase in carbon productivity after the ETS, whereas for others it decreased. Such mixed results were derived primarily because such industries were relatively free from government regulations and less likely to be penalized for their emissions. This also is indicative of heterogeneous effects on industries from ETS implementation. Consistent with prior research, changes in carbon productivity became more stable as the ETS entered a more mature phase^19^.

**Table 1:** Average carbon productivity by industry.

Emission level	Industry classification name	Before ETS	ETS phase 1	ETS phase 2
Top 3	Basic metals	7.938	9.490	10.405
	Chemicals and chemical products	4.695	6.249	8.763
	Other non-metallic mineral products	1.814	6.046	6.238
Bottom 3	Textiles	6.487	4.973	5.204
	Wood and wood/cork products	3.791	5.207	5.678
	Beverages	9.652	8.154	7.150

Industries were classified using the 2-digit Korean Standard Statistical Classification (KSIC) codes (http://kssc.kostat.go.kr/ksscNew_web/ekssc/main/main.do).

**Figure 1:**
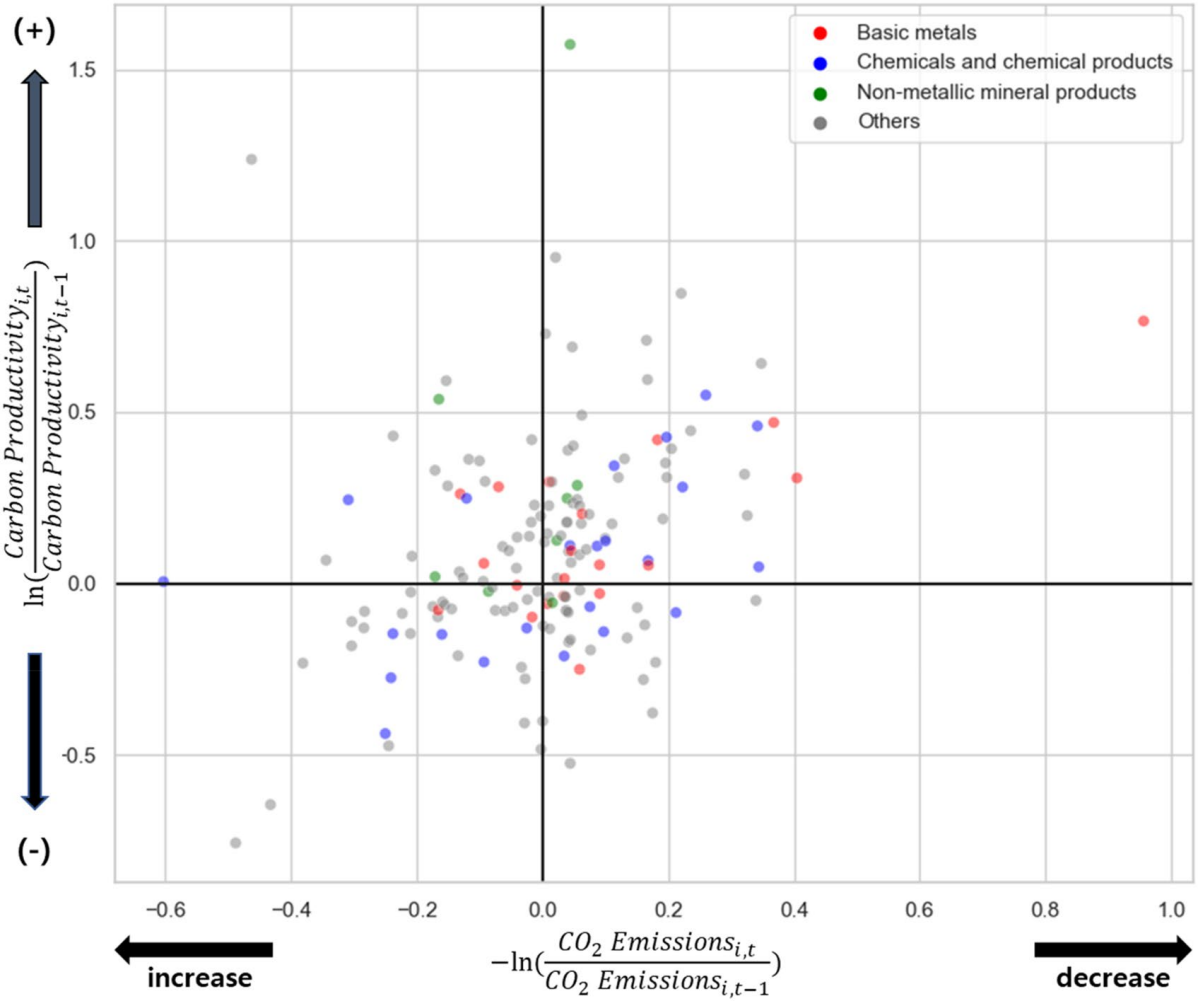
Scatter plot of firm-level changes in carbon emissions and carbon productivity before and after ETS enactment in South Korea. Red indicates the highest emission industry, while green indicates the second highest and blue indicates the third highest.

Figure 1 shows a scatter plot that illustrates how emission reductions contributed to the incremental changes in carbon productivity. The x-axis shows the average log emission change of a firm before and after ETS enactment, and the y-axis shows the average log carbon productivity change before and after ETS enactment. Firms in high-emission industries (colored dots) generally had the largest carbon emission reductions and increased carbon productivity after ETS implementation. For instance, Dongbu Steel reduced its average carbon emissions by 0.04 (log change) while increasing carbon productivity by 1.57 (log change). As for non-metallic mineral products (the second highest-emission industry in South Korea), Asia Cement reduced emissions by 0.95 (log change) while increasing its productivity by 0.77 (log change). The firm-level emission information is from the Ministry of Environment’s Emission Trading Registry System (ETRS) and Offset Registry System. The ETRS annually updates the list of firms participating in ETS and their emission status. Furthermore, the firm-level revenue information is retrieved from the COMPUSTAT database. The data source is a commonly used to gather firm-level information in finance and accounting research area. While we find that the ETS implementation led some manufacturing firms to decrease emissions and increase productivity, especially in high-emission industries, we also find that many firms’ emissions levels increased, and carbon productivity decreased. This implies that the ETS has not engaged all firms to decrease the emissions as designed.

**Figure 2:**
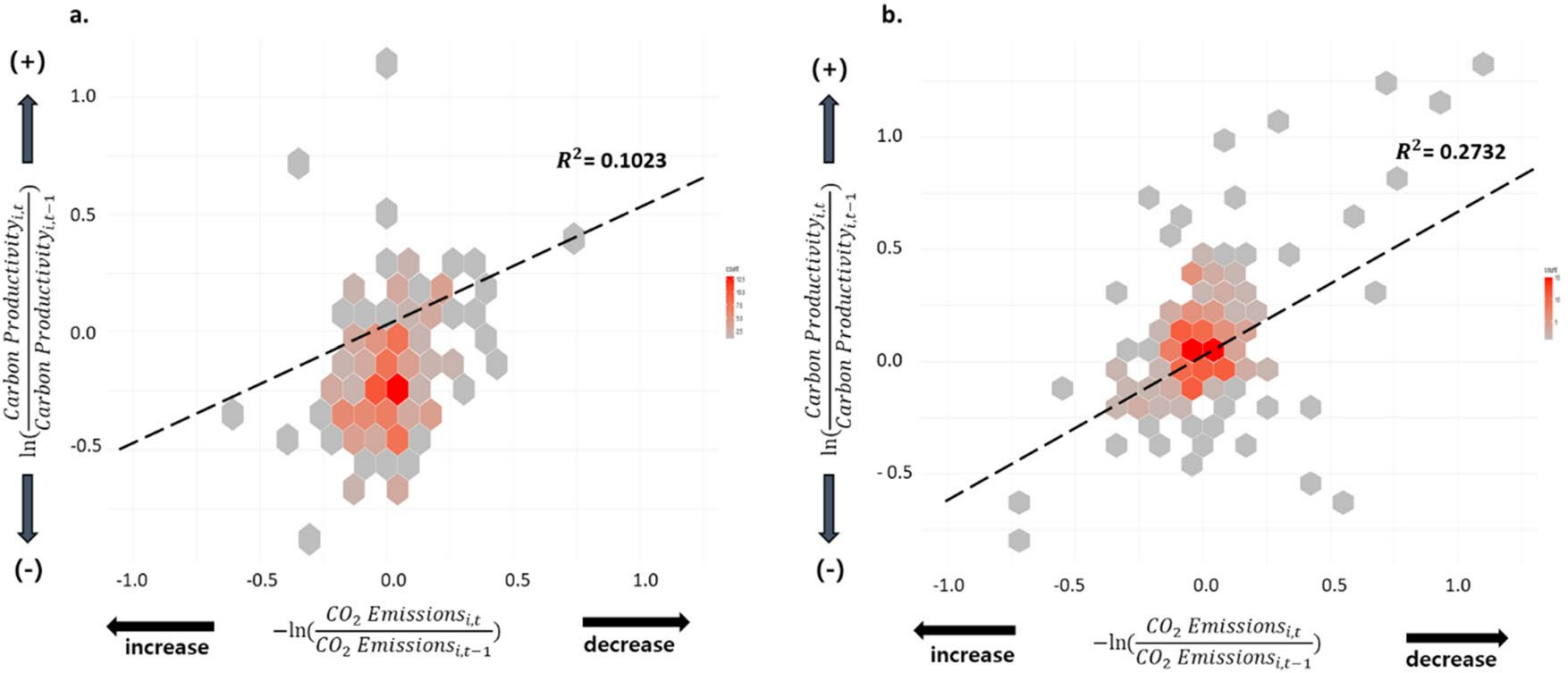
Heatmaps of sample firms’ carbon productivity change by emission level. (**a**) Before ETS to ETS Phase 1. (**b**) ETS Phase 1 to ETS Phase 2. Color scale indicates the number of firms per bin.

Heat maps of changes in firms’ carbon productivity and emissions for two periods, pre-ETS to ETS Phase 1 (Fig. 2a) and ETS Phase 1 to Phase 2 (Fig. 2b), showed that firm-level emissions decreased, and carbon productivity increased overall as the ETS matured. There was a significant increase in carbon productivity or reductions in carbon emissions for some firms during the second ETS phase, but the level of changes varied across firms, thus implying the existence of heterogeneity among ETS participating firms. Furthermore, the heat map in Fig. 2b showed a more linear trend than that in Fig. 2a. The R-squared value of the relationship in the pre-ETS to ETS Phase 1 period was 0.1023, but the value almost doubled from Phase 1 to Phase 2 (0.2732). This indicates that the relationship between emission reductions and carbon productivity became more linear, and that while some firms increased their carbon productivity by decreasing the emissions, some firms experienced increases in the emissions and decreases in the carbon productivity. In other words, more number of firms are beginning to consider reducing the carbon emission while increasing the productivity. The findings that the ETS has become more mature are consistent with previous research on the EU ETS^19^. As interpretations of the univariate analysis results resulted in several endogeneity issues, we employed pooled ordinary least squares (OLS) estimations including control variables and fixed effects to further test our hypothesis.

**Figure 3:**
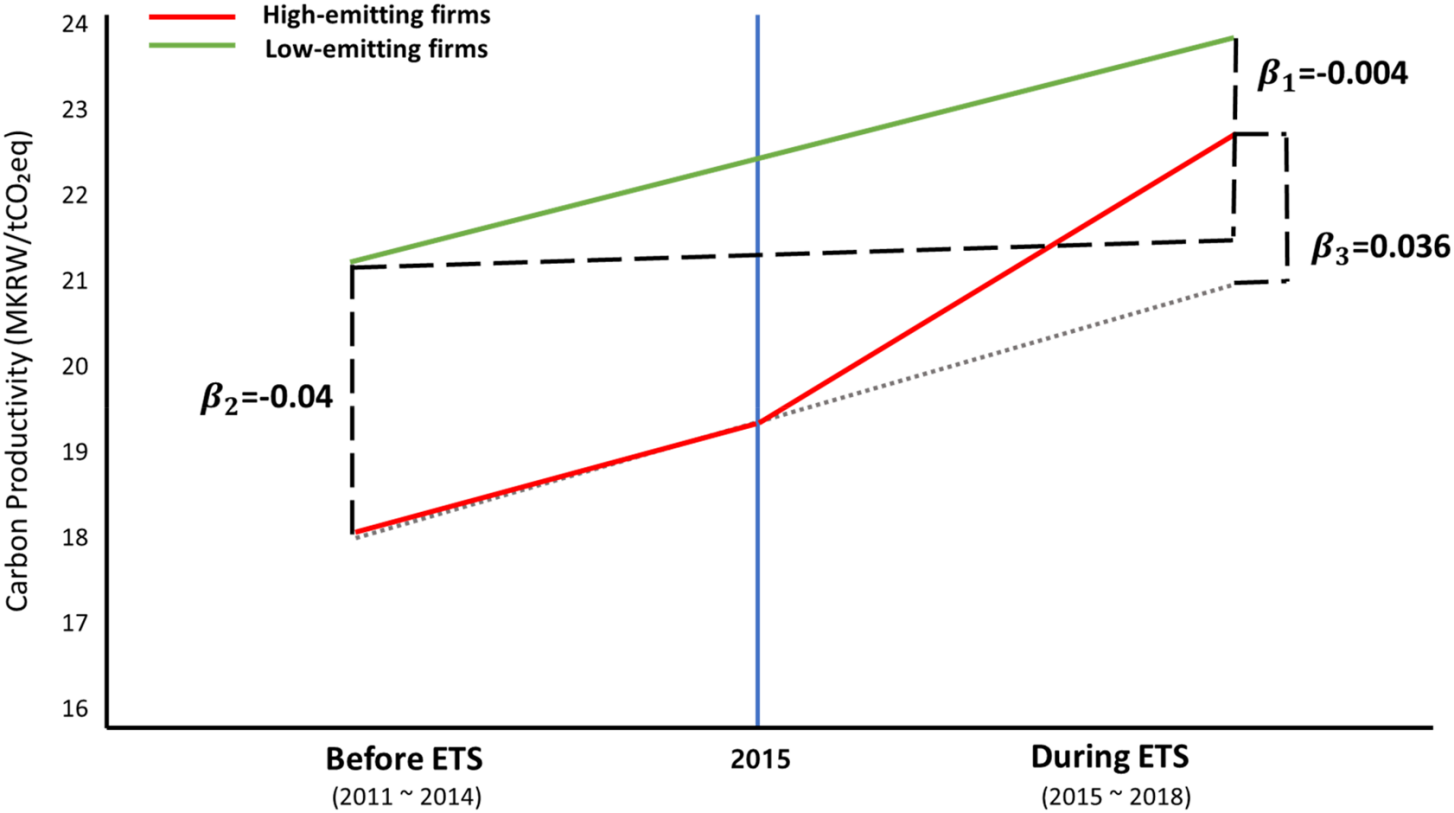
Difference-in-difference results from the baseline estimation model. Beta values are coefficients from Column 3 of Table 2.

The baseline regression results (columns 1–3, Table 2) showed that regardless of the specifications used, carbon productivity of firms in highly emitting businesses tended to be higher for firms participating in the ETS compared to those that did not. In terms of the economic magnitude, carbon productivity was 5.6% higher for ETS participating firms in high-emission industries compared to other group firms (column 3). Figure 3 reports the graphical illustration of the difference-in-difference model results reported in column 3 of Table 2.

**Table 2:** Estimated effects of ETS participation on carbon productivity as determined by regression analyses.

Variable	Original sample			Matched sample		
	(1)	(2)	(3)	(4)	(5)	(6)
	Carbon productivity	Carbon productivity	Carbon productivity	Carbon productivity	Carbon productivity	Carbon productivity
ETS * industry	0.038* (1.763)	0.036* (1.656)	0.036* (1.758)	0.058*** (2.862)	0.055*** (2.751)	0.058*** (3.116)
ETS	0.006 (0.350)	0.003 (0.128)	− 0.004 (− 0.154)	− 0.029* (− 1.948)	− 0.016 (− 0.651)	− 0.014 (− 0.601)
Industry	− 0.017 (− 0.389)	− 0.036 (− 0.773)	− 0.040 (− 0.908)	− 0.028 (− 0.649)	− 0.043 (− 0.954)	− 0.051 (− 1.222)
Constant	0.076*** (2.973)	0.072** (2.285)	− 0.035 (− 1.075)	0.107*** (4.341)	0.088*** (2.891)	− 0.032 (− 1.030)
Observations	995	995	995	1155	1155	1155
Adjusted R-squared	0.118	0.121	0.211	0.142	0.144	0.269
Controls	No	No	Yes	No	No	Yes
Year FE	No	Yes	Yes	No	Yes	Yes
Industry FE	No	Yes	Yes	No	Yes	Yes

Columns 1–3 provide results for non-matched firms, and columns 4–6 provide estimation results for firms paired by propensity score matching (PSM). Columns 1 and 4 report results without control variables and fixed effects (FE), columns 2 and 5 report results with year- and industry-fixed effects, and columns 3 and 6 includes control variables. Parentheses report t-values based on standard errors clustered by firm. *, **, and *** indicate significance at the 10%, 5%, and 1% level, respectively.

The government has selected high-emission firms for participation in the ETS, but these firms are also likely to be large and capable of altering their emissions strategy rapidly, so our results could have been due to unobserved firm-specific factors. To solve this endogeneity issue, we used propensity score matching (PSM), a statistical matching technique that attempts to estimate the effect of a treatment, policy, or other intervention by accounting for the covariates that predict the treatment’s reception^26^. The PSM method attempts to reduce the bias from confounding variables that can be found in an estimate of the treatment effect by simply comparing outcomes among units that received the treatment versus those that did not. Therefore, we employed PSM to match ETS-participating firms with non-participating firms by characteristics. We then re-estimated the regression by using the matched samples (columns 4–6, Table 2). The regression analysis with matched samples was robust and consistent with our hypothesis, thus indicating that our findings were not driven by endogeneity.

Having established that, we next investigated the underlying mechanisms through which manufacturing firms enhanced carbon productivity, such as by modifying manufacturing processes for improved efficiency. The first mechanism is the firm’s profitability level. To achieve the target emission level without decreasing the productivity, firms would need enough resources for facility investment or R&D projects. This leads to the second mechanism, which is the firm’s innovation. As firms with more patents registered annually may have more exposure and interest in green growth, we conjectured a positive relationship between ETS participation and carbon productivity for innovative firms. We then investigated whether board characteristics affected emission strategies. Prior research on corporate governance has shown that managers’ academic or work experience is well-reflected in corporate policies. For example, CEOs that are lawyers tend to improve firms’ information environment^27^, and military CEOs pursue lower corporate investment and are less likely to be involved in fraudulent activity, thereby performing better during industry downturns^28^. As determining optimal emission strategies would require a higher level of knowledge in related fields, CEOs with experience in the environment-related area (“green CEOs”) would theoretically be more likely to contribute to increased carbon productivity by making more efficient emission decisions.

**Figure 4:**
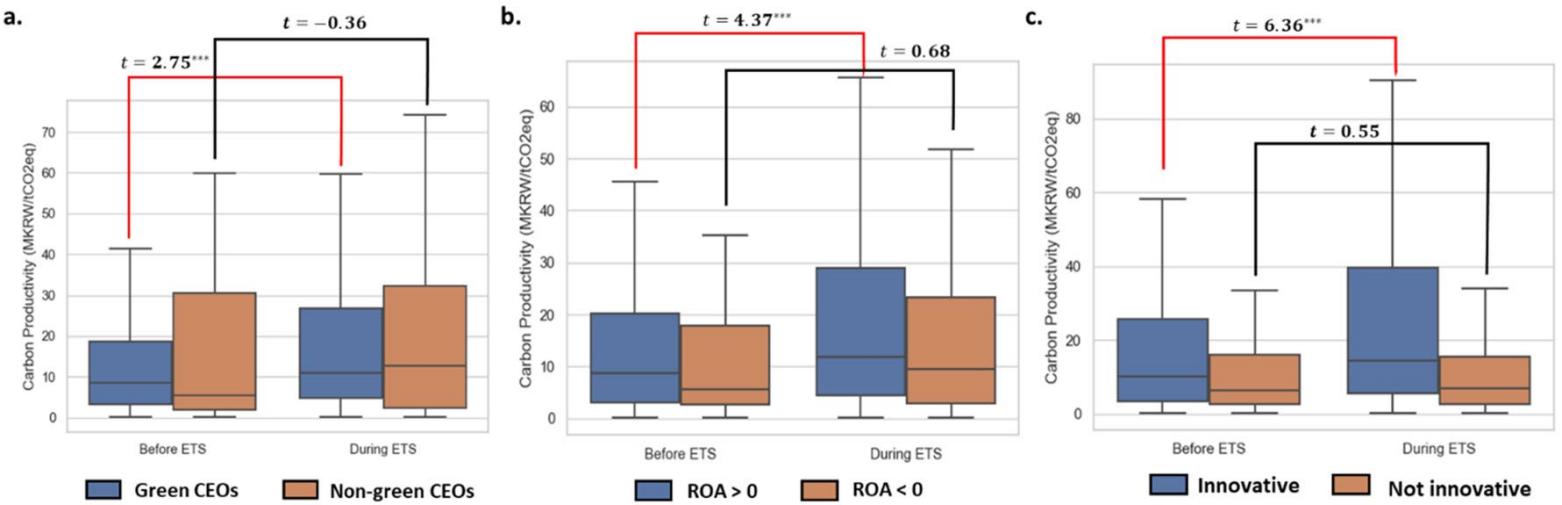
Mechanisms for increasing carbon productivity before and during ETS. Firms are grouped by (**a**) profitability (return on assets, ROA), (**b**) innovation, and (**c**) board characteristics. T-statistics compare carbon productivity values with statistical significance marked as *** for a p-value < 0.01, ** for a p-value < 0.05, and * for a p-value < 0.1.

We assessed changes in carbon productivity for firms grouped by profitability (return on assets, ROA), innovation, and board characteristics (Fig. 4). For the first, we divided our sample firms into profitable (ROA > zero) and not profitable (ROA < zero) groups. For the second, we divided our sample firms into innovative (firm-year observations with > 1 registered patent) and non-innovative (no registered patents) groups. For management characteristics, we divided our sample firms into those managed by green CEOs and those not. The resulting univariate analysis showed that profitable, innovative, and green-CEO-managed firms significantly increased their carbon productivity after joining the ETS. Figure 4 provides the univariate comparison results showing that the firm-level carbon productivity was on average higher for profitable, innovative, and green CEO managing firms, and the findings were statistically significant.

Based on these results, we used the same specification method for the baseline regression to investigate whether such differences could be observed using the difference-in-difference model (Table 3). We again found statistically significant results showing that profitable, innovative, and green-CEO-managed firms in high-emission industries had carbon productivities 4.7%, 2.1%, and 3.3% higher than other firms, respectively, after joining the ETS. The findings demonstrate that to overcome the challenges of an ETS, firms should be less financially constrained so that they have enough resources to invest in innovative projects. Furthermore, managing such projects can be more efficiently driven by green CEOs.

**Table 3:** Estimated effects of firm size, R&D intensity, and board characteristics on carbon productivity as determined by regression analyses.

Variable	ROA > 0	ROA < 0	Patent > 0	Patent = 0	Green CEO	Non-green CEO
	(1)	(2)	(3)	(4)	(5)	(6)
	Carbon productivity	Carbon productivity	Carbon productivity	Carbon productivity	Carbon productivity	Carbon productivity
ETS * industry	0.047** (2.472)	− 0.072* (− 1.665)	0.052* (1.963)	0.022 (0.623)	0.047* (1.805)	− 0.013 (− 0.450)
ETS	− 0.031 (− 1.374)	0.152*** (2.851)	− 0.010 (− 0.289)	0.009 (0.216)	0.001 (0.028)	0.019 (0.499)
Industry	− 0.061 (− 1.521)	0.086 (0.988)	− 0.028 (− 0.522)	− 0.050 (− 0.638)	− 0.044 (− 0.814)	0.012 (0.186)
Constant	0.055** (2.090)	0.021 (0.324)	0.063 (1.553)	0.088* (1.884)	0.080** (2.308)	0.021 (0.406)
Observations	882	113	522	472	801	194
Adjusted R-squared	0.08	0.246	0.038	0.204	0.158	0.199
Controls	Yes	Yes	Yes	Yes	Yes	Yes
Year FE	Yes	Yes	Yes	Yes	Yes	Yes
Industry FE	Yes	Yes	Yes	Yes	Yes	Yes

Parentheses present t-values based on standard errors clustered by firm. *, **, and *** indicate significance at the 10%, 5%, and 1% levels, respectively.

## Discussion and conclusion

ETSs are one of the most popular methods used by governments to reduce GHGs. Departing from the ongoing debates on whether ETSs are effective or not, it is a commonly accepted notion that the ETS market will grow gradually. As the effects of ETS manifest, carbon management is expected to become one of the most important areas of policymaking for a firm, especially if the firm belongs to a high-emission industry. Deployment of market-based mechanisms to reduce carbon emissions creates a burden for companies without a doubt. While it is important to help firms overcome new challenges, guiding firms has been difficult because of the lack of firm-level emission data before the implementation of ETSs worldwide. However, with South Korea’s emission data provided under the Energy and GHG Target Management System, we were able to conduct a firm-level analysis using the emission information before and after the ETS enactment. The results showed that employing a market-based mechanism to reduce emissions encourages firms to alter their manufacturing schemes towards processes with lower GHG emissions.

The fact that carbon productivity has improved in response to the introduction of the ETS seems to be a good sign that South Korean companies can continuously transition to improved environmental management practices in the future. In addition, it is an important implication that innovation is essential in increasing carbon productivity. For this to happen, firms should continue their innovation efforts, which can be potentially achieved by hiring green-minded executives. From the perspective of the government, it is necessary to establish a strategy for supporting and fostering companies seeking to innovate, along with appropriate regulations on high carbon emission companies.

Thus, we believe the results of this research will help inform managerial decisions on carbon. The study provides empirical evidence that the profitability, innovation level, and availability of environmental expertise within a firm can have significant explanatory power in understanding the mechanism behind the effects of an ETS on carbon productivity.

## Methods

### Measuring carbon productivity

We gathered annual emissions data for firms regulated under the 2010 Framework Act on Low Carbon, Green Growth from the Ministry of Environment’s Emission Trading Registry System (ETRS) and Offset Registry System. The ETRS annually updates the list of firms participating in ETS and their emission status. We matched pre- and post-ETS firms with codes from the Korean Exchange (KRX). Using the resulting data, we calculated the carbon productivity as follows:

1 $${CP}_{i,t}=\frac{{Revenue}_{i,t}}{{Emission}_{i,t}},$$

where ${CP}_{i,t}$ is the carbon productivity of firm $i$ in year $t$, ${Revenue}_{i,t}$ is the annual sales generated by firm $i$ in year $t$, and ${Emission}_{i,t}$ is the level of GHGs emitted by firm $i$ in year $t$. For clarity, we multiplied all of the carbon productivity values by one million.

We also included several control variables to address potential unobserved heterogeneity, and these were retrieved by using the COMPUSTAT database. The variables used are as follows: log of the total assets, Tobin’s Q, log of the sales, asset tangibility, cash-to-assets ratio, and cash flow (see Appendix for full definitions and data sources). We then merged the emissions data and other firm-level control variables. Furthermore, we limited the data to manufacturing firms because discussions of carbon productivity apply primarily to manufacturing firms. Notably, it is difficult to compare the carbon productivity of manufacturing industries to other industries where the revenue stream is not largely dependent upon the running of factories. The final dataset spanned 2011–2019 with 1547 firm-year matched panel observations.

### Baseline regression

We first used the difference-in-difference setting to observe the exogenous effects of emission trading scheme (ETS) participation, especially in regard to its effect on firms in high-emission industries, and a pooled ordinary least squares (OLS) regression was used to analyze the effects:

2 $$\begin{aligned} {Carbon \; Productivity}_{j,t} & ={\beta }_{0}+{\beta }_{1}{ETS}_{j,t}*{Industry}_{j,t}+{\beta }_{2}{ETS}_{j,t} \\ & \quad +{\beta }_{3}{Industry}_{j,t}+\sum_{j=1}^{n}{\beta }_{j+3}{CONTROLS}_{j,t}+{\varepsilon }_{j,t}, \end{aligned}$$

where ${Carbon \; Productivity}_{j,t}$ indicates firm-level carbon productivity, ${ETS}_{j,t}*{Industry}_{j,t}$ indicates the main variable of interest where the exogenous entry of the ETS dummy variable interacts with the industry emission dummy variable, ${ETS}_{j,t}$ indicates an indicator variable that equals 1 if the firm participates in ETS and 0 if the firm does not participate in ETS, ${Industry}_{j,t}$ is an indicator variable that equals 1 if the industry to which the firm belonged to emitted > 10,000 t CO_2_-eq annually on average and 0 if below, and ${CONTROLS}_{j,t}$ indicates the set of control variables used in the main regression.

### Propensity score matching (PSM) model construction

Although we included year- and industry-fixed effects, the regression results may still have contained endogeneity issues, so we addressed this issue by using PSM. We assumed two treatments, namely, firms participating in ETS and firms not participating, with *N* independent and identically distributed random variables. Each subject $i$ responded to treatment ${r}_{1i}$ and to control ${r}_{0i}$. We then computed the average treatment effect (the quantity to be estimated) as ${E[r}_{1}]$ − ${E[r}_{0}]$. This produced variable ${Z}_{i}$, where $Z=1$ if subject $i$ was treated and $Z=0$ if subject $i$ was controlled. We defined ${X}_{i}$ as a vector with observed pretreatment covariates for the ${i}$th subject. Although observations of ${X}_{i}$ were pre-determined before the treatment assignment, its features could not include any of the variables used to determine the treatment assignment. The numbering was assumed not to contain any information beyond what was contained in ${X}_{i}$.

For a sample firm with a vector of covariates $X$, and some potential outcomes ${r}_{0}$ and ${r}_{1}$ under the control and treatment, treatment assignment was readily ignorable if the potential outcomes were independent of the treatment conditional on $X$:

3 $${r}_{0},{r}_{1}\perp Z|X.$$

We then formulated the balancing score function $b(X)$, a function of covariates such that the conditional distribution of $X$ given $b(X)$ was identical for treated and controlled samples:

4 $$Z\perp X|b\left(X\right).$$

The most trivial function would be $b\left(X\right)=X$. We finally measured the propensity score, which is defined as the probability of a firm being assigned to ETS participation given a set of other control variables (observed covariates). These were used to reduce selection bias by equating groups based on control variables. Given a binary treatment indicator $Z$ with the control variables $X$, the propensity score was defined as the conditional probability of treatment given *X*:

$$e\left(x\right)\equiv Pr\left(Z=1|X=x\right).$$

We used the following variables as the set of covariates: assets, sales, Tobin’s q, asset tangibility, leverage, cash, and cash flow.

## Data Availability

The data used in the regression analysis are available upon any reasonable requests submitted to the corresponding author. The firm-level carbon emission data are available in the public domain at https://etrs.gir.go.kr/home/index.do?menuId=12.

## Appendix

See Table 4.

**Table 4:** Description and source of the variables analyzed in this study.

Variable name	Description	Source
**Dependent variable**		
Carbon productivity	Sales scaled by tons of CO_2_ equivalent emitted by ETS-participating firms. Values were multiplied by 1 million for clarity	Ministry of Environment/COMPUSTAT
**Independent variable**		
ETS	Indicator variable equaling 1 if the firm participated in ETS	Ministry of Environment
**Subsample variables**		
ROA	Return on assets	COMPUSTAT
Patent	Indicator variable equaling 1 if the firm registered more than one patent	Korea Intellectual Property Rights Information Service
Green CEOs	Indicator variable equaling 1 if the CEO of a firm has educational or job experiences in environment-related fields	TS2000/JOINS
**Control variables**		
Log(asset)	Log of a firm’s total assets	COMPUSTAT
Tobin’s q	Tobin’s q calculated as: (the market value of common stock + book value of preferred stock + (current liabilities – current assets) + long-term debts)/book value of total assets	COMPUSTAT
Log(sales)	Log of a firm’s sales	COMPUSTAT
Tangibility	Net tangible assets calculated as follows: total assets – intangible assets – total liabilities	COMPUSTAT
Cash	Firm’s net cash	COMPUSTAT
Cash flow	Free cash flow calculated as: net income + (depreciation/amortization) – change in working capital – capital expenditure	COMPUSTAT

## Code availability

The original datasets and preprocessed datasets are available from the corresponding author upon any reasonable requests. All regression analyses were conducted with the STATA statistics tool. STATA codes are also available upon any reasonable request.
